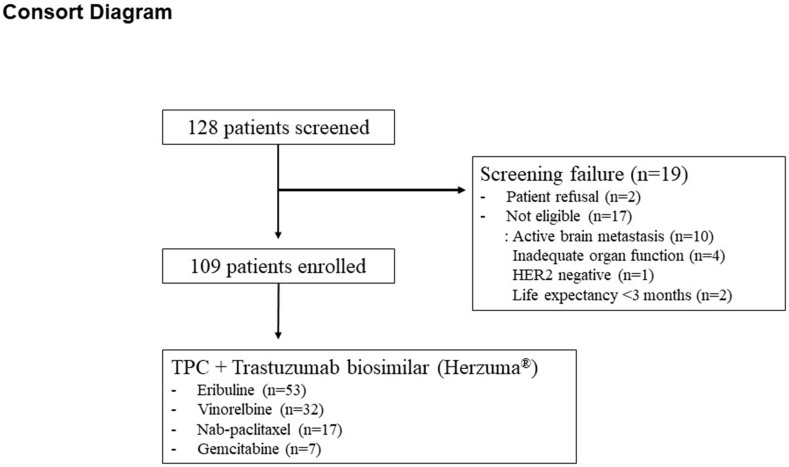# Phase II study to investigate the efficacy of trastuzumab biosimilar (Herzuma®) plus treatment of physician's choice (TPC) in patients with heavily pretreated HER-2+ metastatic breast cancer (KCSG BR 18–14/KM10B)

**DOI:** 10.1016/j.breast.2022.08.002

**Published:** 2022-08-17

**Authors:** Sung Hoon Sim, Jeong Eun Kim, Min Hwan Kim, Yeon Hee Park, Jee Hyun Kim, Koung Jin Suh, Su-Jin Koh, Kyong Hwa Park, Myoung Joo Kang, Mi Sun Ahn, Kyoung Eun Lee, Hee-Jun Kim, Hee Kyung Ahn, Han Jo Kim, Keon Uk Park, Jae Ho Byun, Jin Hyun Park, Gyeong-Won Lee, Keun Seok Lee, Joohyuk Sohn, Kyung Hae Jung, In Hae Park

**Affiliations:** a Center for Breast Cancer, National Cancer Center, Goyang, South Korea; b Department of Oncology, Asan Medical Center, University of Ulsan College of Medicine, Seoul, South Korea; c Division of Medical Oncology, Department of Internal Medicine, Yonsei Cancer Center, Yonsei University College of Medicine, Seoul, South Korea; d Division of Hematology/Oncology, Department of Medicine, Samsung Medical Center, Sungkyunkwan University School of Medicine, Seoul, South Korea; e Department of Internal Medicine, Seoul National University Bundang Hospital, Seoul National University College of Medicine, Seongnam, South Korea; f Department of Hematology and Oncology, Ulsan University Hospital, Ulsan University College of Medicine, Ulsan, South Korea; g Division of Oncology/Hematology, Department of Internal Medicine, Korea University College of Medicine, Anam Hospital, Seoul, South Korea; h Division of Oncology, Department of Internal Medicine, Haeundae Paik Hospital, Inje University College of Medicine, Busan, South Korea; i Department of Hematology-Oncology, Ajou University School of Medicine, Suwon, South Korea; j Department of Hematology and Oncology, Ewha Womans University Hospital, Seoul, South Korea; k Division of Hematology/Medical Oncology, Department of Internal Medicine, Chung-Ang University Hospital, Seoul, South Korea; l Division of Medical Oncology, Department of Internal Medicine, Gachon University Gil Medical Center, Incheon, South Korea; m Division of Oncology and Hematology, Department of Internal Medicine, Soonchunhyang University Cheonan Hospital, Cheonan, South Korea; n Division of Hematology/Oncology, Department of Internal Medicine, Keimyung University Dongsan Hospital, Daegu, South Korea; o Department of Internal Medicine, Incheon St. Mary's Hospital, College of Medicine, The Catholic University of Korea, Seoul, South Korea; p Department of Internal Medicine, Seoul Metropolitan Government Seoul National University Boramae Medical Center, Seoul National University College of Medicine, Seoul, South Korea; q Division of Hematology and Oncology Department of Internal Medicine Gyeongsang National University College of Medicine, Jinju, South Korea; r Division of Oncology/Hematology, Department of Internal Medicine, Korea University College of Medicine, Guro Hospital, Seoul, South Korea

**Keywords:** HER2 positive, Metastatic breast cancer, Trastuzumab biosimilar, Clinical trial, ctDNA

## Abstract

We investigated the efficacy and safety of a trastuzumab biosimilar, Herzuma®, in combination with treatment of physician's choice (TPC) in patients with HER2+ metastatic breast cancer (MBC) who had failed at least two HER2 directed chemotherapies.

## Highlights

• Treatment of HER2-positive breast cancer is rapidly evolving with the development of new agents. However, treatment options are still limited after progression with proven anti-HER2 therapies.
• The combination of chemotherapy with a trastuzumab biosimilar, Herzuma®, was effective and safe in patients with heavily pre-treated HER2+ MBC.
• Duration of response to previous anti-HER2 therapy, *PIK3CA* and *ERBB2* mutations were associated with clinical efficacies of trastuzumab biosimilar plus chemotherapy.

A total of 109 patients were enrolled and received Herzuma® with TPC (gemcitabine, vinorelbine, eribulin, capecitabine, or nab-paclitaxel). Genetic biomarkers were examined using targeted sequencing in patients with available tumor tissues or blood samples (n = 100). Safety and quality of life (QoL) data were collected for all patients.

Patients had received a median of three previous anti-HER2 therapies (range 2–8). The median PFS and OS were 4.6 months (95% CI 2.8–7.2) and 18.6 months (95% CI 14.4-not reached), respectively. Objective response rate was 18.7%. Younger age (<55 years) and liver metastasis were associated with a shorter PFS. A longer response to previous anti-HER2 therapy (>6 months) was associated with PFS improvement. For biomarker analysis, patients with mutations of *PIK3CA* (23%) or *ERBB2* (7.0%) had shorter PFS. Response to previous anti-HER2 therapy (>6 months) (HR = 0.53, 95% CI 0.34–0.82), *PIK3CA* mutations (HR = 2.32, 95% CI 1.38–3.92), and *ERBB2* mutations (HR = 5.18, 95% CI, 2.11–12.72) were significant predictors of PFS by multivariable analysis. Toxicities were mild and manageable, and a grade 3 cardiac event occurred in one patient. The QoL measured by QLQ-30 was maintained during the treatments.

The combination of chemotherapy with a trastuzumab biosimilar, Herzuma®, was effective and safe in patients with heavily pre-treated HER2+ MBC. Duration of response to previous anti-HER2 therapy, *PIK3CA* and *ERBB2* mutations were associated with PFS.

## 1 Introduction

Breast cancer is the most common cancer worldwide in women and human epidermal growth factor receptor 2 (HER2) is overexpressed in approximately 20% of patients [1]. In HER2 positive breast cancer, HER2 targeting chemotherapy is the main treatment strategy.

After the introduction of the first HER2 targeting drug, trastuzumab, many anti-HER2 agents have been developed, and have significantly changed the landscape of treatment for HER2 positive breast cancer [2,3]. The current consensus includes dual anti-HER2 blockade agents (trastuzumab plus pertuzumab) with taxane as the first line treatment for HER2 positive metastatic breast cancer (MBC), followed by fam-trastuzumab-deruxtecan (T-Dxd) or trastuzumab-emtansine (T-DM1) in the second line setting [[4], [5], [6]]. In addition, anti-HER2 tyrosine kinase inhibitors or engineered antibodies may be one of the important options in the treatment line-up [4]. However, considering the limited accessibility of all these drugs, re-administration with trastuzumab and cytotoxic chemotherapy should still be considered an important treatment option [4,7,8].

CT-P6 (Herzuma®, CELLTRION, Incheon, Republic of Korea), a biosimilar of the reference trastuzumab, demonstrated comparable efficacy and safety with trastuzumab in clinical trials for equivalence testing [9]. Although CT-P6, Herzuma®, has already been used in many countries for the same indications as the reference trastuzumab, the clinical efficacy and safety of its re-administration after trastuzumab have not been tested yet.

In this study, we investigated the efficacy and safety of a trastuzumab biosimilar, Herzuma®, in combination with the treatment of physician's choice (TPC) in patients with HER2 positive MBC who had failed 2 or more HER2 directed chemotherapies. We also examined the clinical and genetic factors associated with the clinical benefit of trastuzumab re-administration.

## 2 Patients and methods

### 2.1 Study design and patients

This multi-center, single arm, phase 2 study was conducted by the Korean Cancer Study Group (KCSG, KCSG-BR 18–14/KM10B) in Republic of Korea. Patients aged 19 years or older with histologically confirmed HER2 positive metastatic or unresectable breast cancer were eligible. HER2 positivity was defined as 3+ by immunohistochemical staining or 2+ with positive fluorescence by in situ hybridization (silver in situ hybridization or chromogenic in situ hybridization were accepted) according to the guidelines of the American Society of Clinical Oncology and College of American Pathologists [10]. Additional inclusion criteria were an Eastern Cooperative Oncology Group (ECOG) performance status score of 0–2; adequate heart function with left ventricular ejection fraction (LVEF) ≥ 50%; and adequate organ functions. Patients should have previously received at least two anti-HER2 based chemotherapies for their metastatic diseases. Other eligibility criteria were patients with treatment-related toxicities ≤ grade 2 according to the National Cancer Institute Common Terminology Criteria for Adverse Events (NCI-CTCAE) v 5.0 and measurable or evaluable lesions according to the Response Evaluation Criteria in Solid Tumors version 1.1 (RECIST v 1.1). Patients with symptomatic brain metastases and serious medical problems such as heart failure, uncontrolled diabetes, or infection were excluded.

The primary endpoint of this study was progression-free survival (PFS), and the secondary endpoints were the objective response rate (ORR), overall survival (OS), safety, and quality of life (QoL).

This study was done in accordance with the guidelines for Good Clinical Practice and the Declaration of Helsinki and was approved by the institutional review board. All study procedures were conducted after receiving written informed consent from each participant.

### 2.2 Treatment and assessment

The patients received a trastuzumab biosimilar (Herzuma®) on cycle 1 day 1 at 8 mg/kg as a loading dose, then 6 mg/kg every 3 weeks. TPC was chosen by each investigator among gemcitabine, vinorelbine, eribulin, capecitabine, or nab-paclitaxel. The treatment cycle for TPC was 3 weeks and its dosage was adjusted at the discretion of the investigators. The treatment continued until disease progression, unacceptable toxicity, or patient withdrawal.

Tumors were assessed using RECIST v 1.1 at screening and every 6 weeks from the initiation of treatment. Adverse events were evaluated and recorded according to NCI-CTCAE v 5.0 at baseline and throughout treatment. Assessment of left ventricular ejection fraction were performed at baseline, every 9 weeks during the treatment period, and at the end of treatment visit by 2D-echocardiogram. The QoL was measured by the European Organization for Research and Treatment Care Quality of Life Questionnaire (Korean version, EORTC QLQ-C30) at baseline, after 2 cycles, and at the end of treatment.

### 2.3 Biomarker analyses

During the screening period, tumor tissues (primary or metastatic FFPE tumor tissue) or blood samples were obtained for exploratory biomarker analysis. Targeted sequencing based on next generation sequencing was carried out with the K-MASTER project, a Korean solid cancer genome analysis research project [11]. The targeted sequencing using tumor tissues was performed using CancerSCAN™ (Samsung Genome Institute, Seoul, Korea). In patients whose tumor tissues were not available, 10 mL of whole blood was collected with Cell-Free DNA BCT® for ctDNA preparation and analyzed by the Axen™ Cancer Panel (Macrogen, Seoul Korea).

### 2.4 Statistical analysis

We hypothesized that the median PFS of the study population would be 3.8 months compared with 2.8 months in the historical control group (chemotherapy only group in the TH3RESA trial) [12]. To test this hypothesis with one sided type I error rates of 0.1 and 80% power, a total of 108 patients was needed. The PFS and OS were estimated using the Kaplan-Meier method. In an exploratory biomarker study, the PFS was analyzed for each biomarker subgroup using the Kaplan-Meier method and compared by the unstratified log-rank test. Uni- and multivariable analyses were performed using the Cox proportional hazard model. The ORR was the percentage of patients who were determined to have an objective response [complete response (CR) + partial response (PR)] based on RECIST v 1.1. For the ORR analysis, patients who did not have any record of post-baseline tumor assessment were counted as non-responders. Safety analyses was performed on the treatment population who received any amount of study treatment. The EORTC-QLQ-C30 data were scored according to the EORTC-QLQ-C30 scoring manual [13]. Summary statistics of absolute scores of the QLQ-C30 scales and the changes from baseline were summarized. Only patients with baseline assessment and at least one post-baseline assessment were included in this analysis. Statistical analyses were done with Rstudio, version 4.1.1. This study was registered with ClinicalTrials.gov, number NCT03755141.

## 3 Results

### 3.1 Patient characteristics

Between December 2018 and February 2021, 128 patients were enrolled and followed up. Nineteen patients failed the screening procedure (Consort diagram). Finally, 109 patients received the study treatment and were included in the efficacy and safety analyses. Baseline patient characteristics were presented in Table 1. The median age was 55 years (range, 20–76) and 59 patients (54.1%) had hormone receptor positive tumors (Table 1). TPCs consisted of eribulin (48.6%), vinorelbine (29.4%), nab-paclitaxel (15.6%), and gemcitabine (6.4%). Patients had received a median of three prior anti-HER2 therapies (range 2–8) and 66 patients (60.6%) had received more than three lines of chemotherapies for their metastatic breast cancer. The duration of response to anti-HER2 therapy administered immediately prior to study enrollment was ≤6 months in 54 (49.5%), >6 and ≤ 12 months in 28 (25.7%), and >12 months in 27 patients (24.8%).

**Table 1:** Patient characteristics.

	N = 109
Age (years, median, range)	55 (20–76)
Menstruation status	
Premenopausal	32 (29.6%)
Postmenopausal	76 (70.4%)
ER and PgR status	
ER/PgR (+)	59 (54.1%)
ER/PgR (−)	48 (44.0%)
Unknown	2 (1.8%)
ECOG PS	
1	32 (29.4%)
2	77 (70.6%)
De novo stage IV	34 (31.2%)
TPC regimens	
Eribuline	53 (48.6%)
Vinorelbine	32 (29.4%)
Nab-paclitaxel	17 (15.6%)
Gemcitabine	7 (6.4%)
Metastatic sites	
Lung	75 (68.8%)
Liver	41 (37.6%)
Brain	21 (19.3%)
No. of prior chemotherapy regimens at metastatic setting*	
1-3	43 (39.4%)
>3	66 (60.6%)
No. of prior anti-HER2 therapy at metastatic setting**	
1-3	83 (76.1%)
>3	26 (23.9%)
Prior anti-HER2 agents	
Trastuzumab Pertuzumab Trastuzumab emtansine (T-DM1)	108 (99.1%) 45 (41.3%) 107 (98.2%)
Lapatinib	81 (74.3%)
Trastuzumab deruxtecan (T-DXd)	12 (11.0%)
Duration of response to previous anti-HER2 therapy***	
≤6 months	54 (49.5%)
>6 & ≤12 months	28 (25.7%)
>12 months	27 (24.8%)

ER, estrogen receptor; PgR, progesterone receptor; ECOG PS, European Cooperative Oncology Group Performance Status; TPC, treatment of physician's choice; *, Number of all administered chemotherapies regardless of anti-HER2 drugs at metastatic setting; **, Number of anti-HER2 based treatment at metastatic setting; ***, duration of response to anti-HER2 therapy administered immediately prior to study enrollment.

### 3.2 Efficacy and biomarker analysis

The median follow-up duration was 13.3 months (95% CI, 10.8–14.6). Nine patients were still on the trial at the time of the data cutoff (September 2021). The median number of cycles of TPCs with trastuzumab biosimilar doses was 6 (range 1–30). The main reason for treatment discontinuation was disease progression. The ORR was 18.7% (CR = 0, PR = 20). Thirty-three patients (30.3%) showed stable disease for more than 24 weeks. The median PFS was 4.6 months (95% CI 2.8–7.2), surpassing our hypothesized PFS of 3.8 months (Fig. 1A). The median OS was 18.6 months (95% CI 14.1 – not reached) (Fig. 1B). According to a clinical subgroup analysis, the median PFS was significantly improved in patients ≥55 years (5.6 vs. 4.2 months, HR = 0.62, 95% CI 0.42–0.92, p = 0.019) and with a response duration to previous anti-HER2 therapy >6 months (5.8 vs. 3.3 months, HR = 0.52, 95% CI 0.35–0.78, p = 0.001) (Fig. 1C and D). Patients with liver metastasis showed a trend of shorter PFS (HR = 1.47, 95% CI 0.98–2.21. p = 0.066). Other clinical factors including ECOG PS, hormone receptor status, and number of prior chemotherapies or anti-HER2 therapy, were not associated with PFS (data not shown).

**Fig. 1:**
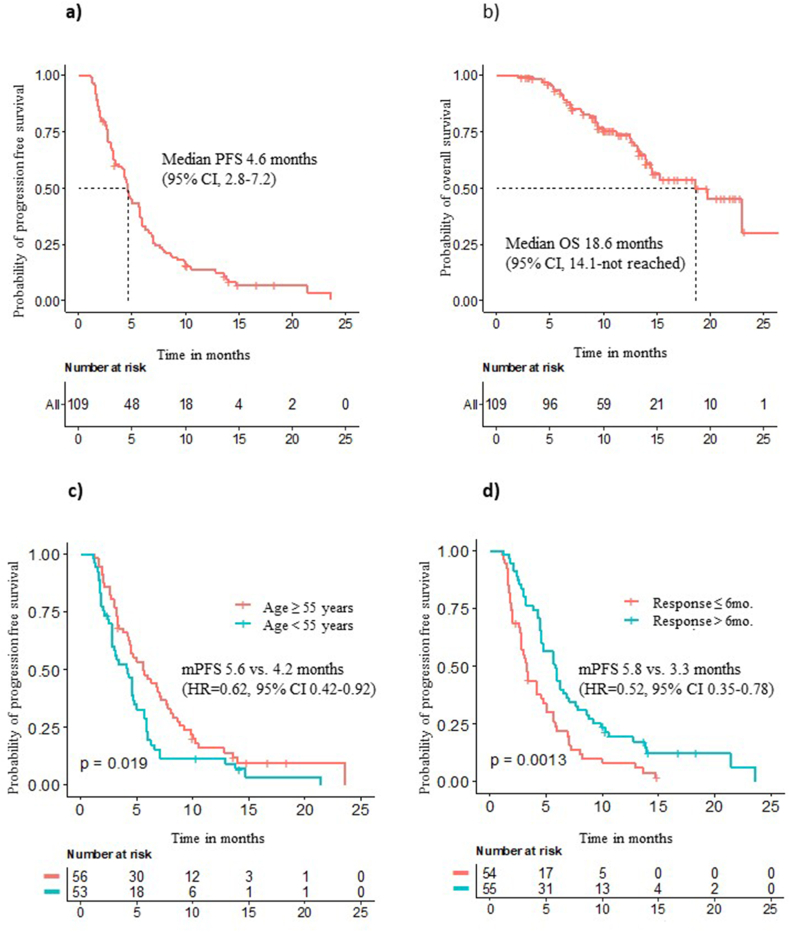
Kaplan-Meier curves of progression free survival and overall survival. a) Progression free survival in all patients, b) Overall survival in all patients, c) PFS according to age, d) PFS according to previous response duration to anti-HER2 therapy.

For an exploratory biomarker study, patients provided FFPE tumor tissues (primary = 33, metastatic = 15) or peripheral blood for ctDNA collection if tumor tissues were not available (n = 52). Because samples and test panels were different, using full sequencing data was not suitable for biomarker analysis. Therefore, only the most common gene alterations, *TP53*, *PIK3CA*, and *ERBB2* were selected to investigate the association with PFS. The overall *PIK3CA* mutation rate was 23% (35.4% in tumor tissue, 11.5% in ctDNA), 41% for *TP53* (50% in tumor tissue, 32.7% in ctDNA), and 7.0% for *ERBB2* (8.3% in tumor tissue, 5.8% in ctDNA) (Table 2). Overall, patients with *PIK3CA* mutations (HR = 2.0, 95% CI 1.2–3.3, p = 0.005) or *ERBB2* mutations (HR = 5.6, 95% CI 2.4–13.2, p < 0.001) had significantly shorter PFS (Table 2). This association was more pronounced when analyzed with ctDNA rather than tumor tissue (Table 2).

**Table 2:** Biomarker status and its association with PFS in study participants.

	N (%)	HR (95% CI)	P	
FFPE tumor tissue (n = 48)				
*PIK3CA*	17 (35.4%)	1.76 (0.93–3.33)	0.083	
*TP53*	24 (50.0%)	1.32 (0.73–2.40)	0.359	
*ERBB2*	4 (8.3%)	1.56 (0.69–3.54)	0.284	
**ctDNA (n = 52)**				
*PIK3CA*	6 (11.5%)	5.39 (2.08–13.98)	0.001	
*TP53*	17 (32.7%)	1.83 (0.98–3.44)	0.059	
*ERBB2*	3 (5.8%)	4.4 (1.3–15.4)	0.021	
**Total (n = 100)**				
*PIK3CA*	23 (23.0%)	2.04 (1.25–3.33)	0.005	
*TP53*	41 (41.0%)	1.48 (0.97–2.25)	0.071	
*ERBB2*	7 (7.0%)	5.62 (2.39–13.22)	<0.001	

PFS, progression free survival; FFPE, formalin fixed paraffin embedded; ctDNA, circulating tumor DNA.

In multivariable analysis, the response duration to previous anti-HER2 therapy >6 months (HR = 0.53, 95% CI 0.34–0.82, p = 0.004), *PIK3CA* mutations (HR = 2.32, 95% CI 1.38–3.92, p = 0.002), and *ERBB2* mutations (HR = 5.18, 95% CI 2.11–12.72, p < 0.001) remained significant factors for PFS when adjusted with liver metastasis, age, and hormone receptor status (Fig. 2).

**Fig. 2:**
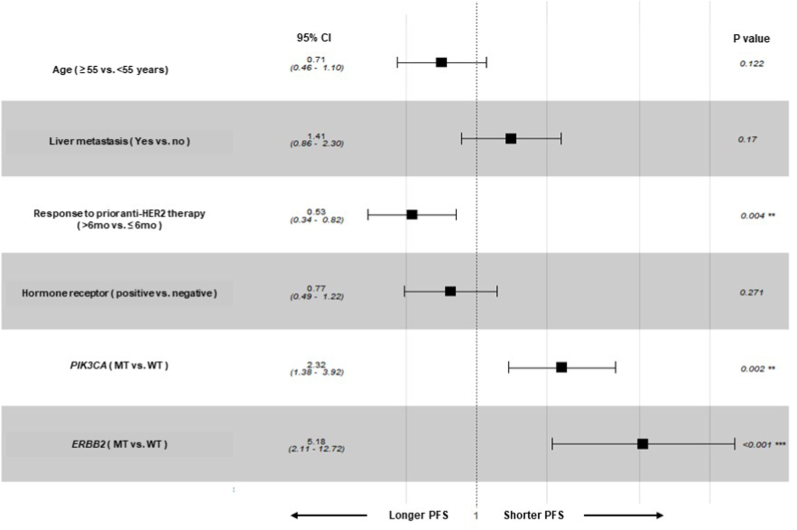
Forest plot of PFS according to clinical and genetic factors.

### 3.3 Safety and quality of life

**Fig. 3:**
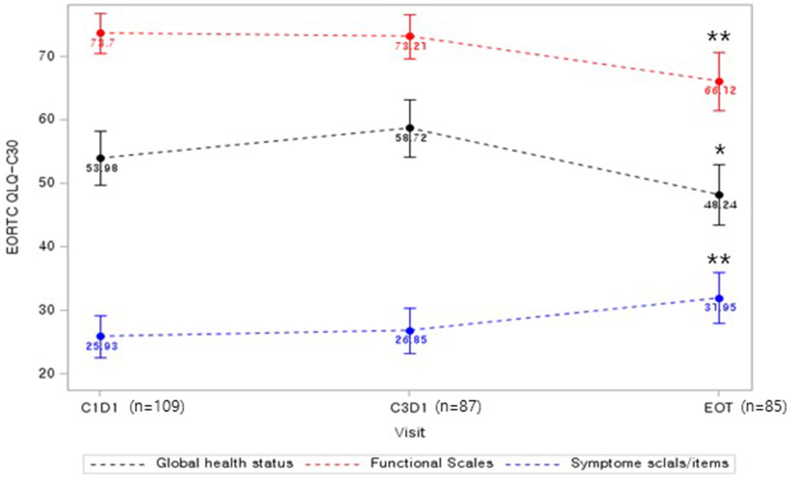
Quality of life analysis in the study population using EORTC-QLQ-C30 questionnaire. EOT, end of treatment; *, P < 0.05; **, P < 0.001.

During the study treatment, the most common adverse events (AE) were hematologic toxicities including neutropenia, thrombocytopenia, and anemia (Table 3). Heart failure (grade 3), decreased LVEF (grade 2), and diastolic dysfunction (grade 2) were reported in one patient each (Table 3). Of the patients who developed cardiac AEs, two discontinued the study treatment permanently. All reported cardiac AEs recovered without any major sequelae. Overall, most of toxicities were mild and tolerable. Regarding the QoL, the questionnaire completion rate was 78.0% at the end of treatment. All three QLQ-C30 scores remained similar at C1D1 (baseline) and C3D1 time points (Fig. 3). However, statistically significant decreases of global health status and functional scales were found at the end of treatment (Fig. 3, Supplementary Table 1). Symptom scales were also increased at the end of treatment, and it seemed to be associated with disease progression (Supplementary Table 1, Supplementary figure 1).

**Table 3:** Safety profiles.

	Grade 1	Grade 2	Grade 3	Grade 4
Hematology				
Neutropenia	4 (3.7%)	9 (8.3%)	15 (13.8%)	9 (8.3%)
Thrombocytopenia	0	2 (1.8%)	0	0
Anemia Febrile neutropenia	0 NA	2 (1.8%) NA	4 (3.7%) 11 (10.1%)	0 0
**Liver function abnormality**				
ALT elevation	4 (3.7%)	2 (1.8%)	1 (0.9%)	0
AST elevation	3 (2.8%)	1 (0.9%)	1 (0.9%)	0
**Cardiac adverse event**				
Heart failure	0	0	1 (0.9%)	0
LVEF decreased	0	1 (0.9%)	0	0
Diastolic dysfunction	0	1 (0.9%)	0	0

ALT, Alanine aminotransferase; AST, Aspartate aminotransferase; LVEF, left ventricular ejection fraction.

## 4 Discussion

This prospective study suggested that a combination of chemotherapy with a trastuzumab biosimilar, Herzuma®, was effective and safe in patients with heavily pre-treated HER2 positive MBC. Treatment with the trastuzumab biosimilar and TPC led to ORR of 18.5%, and 30.3% of patients showed stable disease for 24 weeks or longer. The median PFS and OS were 4.6 months and 18.6 months, respectively. A grade 3 cardiac event occurred in one patient. The PFS was longer in patients with > 6-month response duration of previous anti-HER2 therapy. Biomarker analysis showed mutations of *PIK3CA* and *ERBB2* were associated with shorter PFS.

Continued anti-HER2 based therapy is the current clinical standard for patients with HER2-positive tumors. Therefore, trastuzumab beyond progression should be considered for patients when other anti-HER2 therapies have been exhausted or are not available [14]. In a retrospective study that evaluated the clinical outcomes of patients retreated with trastuzumab-based therapy after disease progression on lapatinib-based treatment, ORR of 29% was observed in patients assessable for response. The median PFS was 4.9 months, and the median OS was 19.4 months [15]. In the SOPHIA trial, which demonstrated an improvement in survival with margetuximab (an Fc-engineered HER2-targeted antibody) plus chemotherapy compared with trastuzumab plus chemotherapy after progression on ≤ 3 line of prior metastatic therapy, the median PFS and OS were 4.9 and 19.8 months in the control group [16]. In our study, 60.6% of patients had been treated with more than 3 lines of prior chemotherapy in a metastatic setting and most patients received prior anti-HER2 treatment with T-DM1 or lapatinib. For such heavily pre-treated HER2-positive breast cancer patients, chemotherapy with a trastuzumab biosimilar, Herzuma® may still be a viable option, as it has shown similar efficacies and safety profiles to previous studies.

We investigated the predictive factors of trastuzumab re-administration in terms of continuing anti-HER2 therapy. As a result, a longer duration of response to previous anti-HER2 therapy (>6 months) was a good predictor of PFS in our study population. In contrast, the number of anti-HER2 therapies or chemotherapies were not associated with PFS. Our study results suggested that if the tumor responded well to previous anti-HER2 therapy, continuing anti-HER2 treatment would be clinically beneficial regardless of the number of drugs administered.

Many clinical studies have reported *PIK3CA* mutations are a potential resistance mechanism to anti-HER2 therapies [17]. In neoadjuvant trials, the presence of *PIK3CA* mutations was associated with a lower rate of pathological CR [18]. In a metastatic setting, *PIK3CA* mutations were associated with a worse prognosis in patients treated with a standard anti-HER2 treatment [19,20]. Similar to previous studies, *PIK3CA* mutations were related to poor PFS in our study population. Several strategies targeting the PI3K pathway have been tried in HER2 positive breast cancer. Previous studies showed that HER2 positive MBC having *PIK3CA* mutations or *PTEN* loss could benefit from mTOR inhibitor, everolimus by blocking activated PI3K-mTOR pathway [21]. Another drug targeting *PIK3CA* mutation directly, Alpelisib has proved its anti-tumor efficacy with endocrine therapy in ER + MBC with *PIK3CA* mutations [22]. Now, it is being tested in HER2 positive MBC with *PIK3CA* mutations in multiple clinical trials and emerging results will guide us to deal with HER2 positive breast cancer with *PIK3CA* mutations (ClinicalTrials.gov Identifier: NCT05063786, NCT05230810, NCT04208178).

*ERBB2* somatic mutations are observed in 2–5% of primary breast cancers and most have been reported in HER2-negative breast cancers [[23], [24], [25]]. However, in a recent study evaluating *ERBB2* mutations using ctDNA in patients with advanced breast cancer, the frequency of *ERBB2* mutations was 8.9% and this was higher in HER2-positive tumors than in HER2-negative tumors [26]. It has been suggested that *ERBB2* mutations are acquired as a consequence of anti-HER2 therapy, which can easily be detected by ctDNA analysis [26,27]. In addition, *ERBB2* mutations were associated with shorter PFS in HER2-positive patients who received trastuzumab plus chemotherapy in a metastatic setting. In our study, *ERBB2* mutations were detected in 7 patients including 2 (6.1%) in primary tumor tissues, 2 (13.3%) in metastatic tumor tissues, and 3 (5.8%) by ctDNA analysis. Although the absolute number of *ERBB2* mutations was small, more were detected in metastatic tumor tissues and were significantly associated with shorter PFS. Interestingly, these genetic alterations were stronger predictors of response to anti-HER2 therapy compared with various clinical factors in heavily pre-treated HER2 positive MBC patients. Therefore, these mutations could be good targets to improve the clinical efficacy of anti-HER2 therapy.

Our study had several limitations. First, it was a nonrandomized, single-arm study design with a small sample size. We set the control group of TH3RESA trial as a historical control and designed our study based on its data [12]. Although direct comparison was not possible, we compared patient characteristics of two trials (Supplementary Tables 2 and 3). Among various clinical factors, our patients showed worse performance and experienced more diverse anti-HER2 therapies before enrollment. The composition of TPC regimen was very different between two groups. These differences can eventually lead to differences in results. Second, because more than 50% of patients were diagnosed with metastatic HER2 positive breast cancer before pertuzumab was available, they were unable to receive pertuzumab which is now considered the standard therapy given with trastuzumab. However, almost all patients have exposed to subsequent therapies including T-DM1 and lapatinib. Therefore, our patient population can be representative of heavily pretreated HER2-positive breast cancer patients, and it is meaningful to see the effect of trastuzumab biosimilar and chemotherapy combination in this group. Third, biomarker analysis was performed using tissue or blood samples due to limited sample availability. Because of the discrepancy between samples and test methods, genetic alterations that were consistently and frequently detected were selected and analyzed. Biomarkers from our study results need to be validated in a large cohort in the future.

In summary, our study showed the clinical efficacy and safety of a trastuzumab biosimilar plus chemotherapy, even in heavily pretreated HER2-positive MBC patients. The response duration of previous anti-HER2 treatment was an important clinical factor for the prediction of clinical efficacy in this population. *PIK3CA* and *ERBB2* mutations were associated with shorter PFS and these markers should be validated in future studies.

## Funding information

This research was supported by a grant of the Korea Health Technology R&D Project through the 10.13039/501100003710Korea Health Industry Development Institute (KHIDI), funded by the 10.13039/100008903Ministry of Health & Welfare, Republic of Korea (grant number: HI17C2206) and the National R&D Program for Cancer Control through the National Cancer Center (NCC) funded by the Ministry of Health & Welfare, Republic of Korea (grant number: HA22C0012).

## Supply of drugs

Herzuma (trastuzumab biosimilar, Celltrion) was provided by Celltrion, South Korea.

## Data collection and analysis

Celltrion did not involve in collecting and analyzing clinical data of this study. All authors had full access to all the data in the study and had final responsibility for the decision to submit for publication.

## Ethics approval and consent to participate

This study was done in accordance with the guidelines for Good Clinical Practice and the Declaration of Helsinki and was approved by the institutional review board. All study procedures were conducted after receiving written informed consent from each participant. This study was registered with ClinicalTrials.gov, number NCT03755141.

## Author contributions

Conception and design: I.H.P, Provision of study material or patients: SHS, JEK, MHK, YHP, JHK, KJS, SK, KHP, MJK, MSA, KEL, HJK, HKA, HJK, KUP, JHB, JHP, GWL, KSL, JS, KHJ, and IHP. Collection and assembly of the data: SHS, JEK, MHK, YHP, JHK, KJS, SK, KHP, MJK, MSA, KEL, HJK, HKA, HJK, KUP, JHB, JHP, GWL, KSL, JS, KHJ, and IHP. Data analysis and interpretation: SHS, JEK, and IHP., Paper writing: SHS, JEK, and IHP., Final approval of the paper: all authors.

## Declaration of competing interest

The authors declare no competing interests except In Hae Park who was provided Herzuma from Celltrion, Korea for this study.

## Figures and Tables

**figs1:**
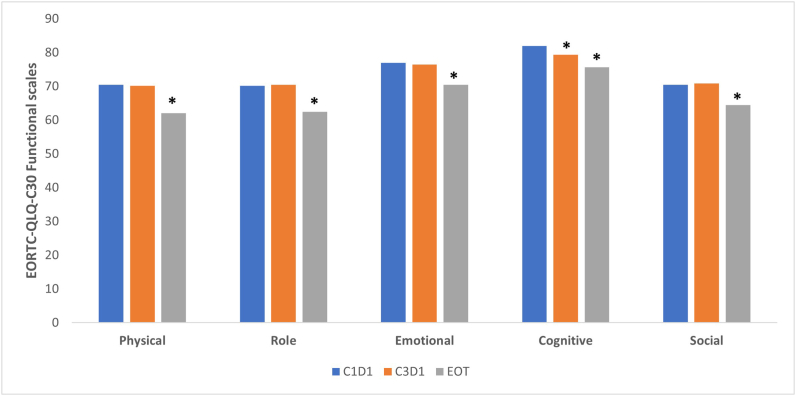


**figs2:**
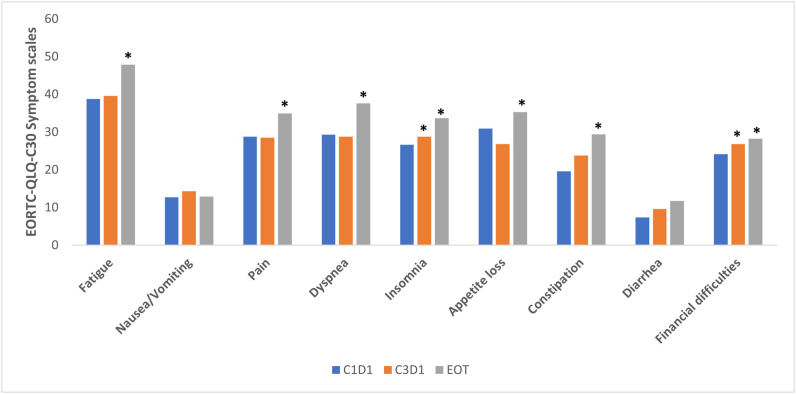


**figs3:**