# Supplementary material for: Phase II study to investigate the efficacy of trastuzumab biosimilar (Herzuma®) plus treatment of physician's choice (TPC) in patients with heavily pretreated HER-2+ metastatic breast cancer (KCSG BR 18–14/KM10B)
Source: Breast. 2022 Aug 17;65:172–8. doi: 10.1016/j.breast.2022.08.002 (PMC9429798; doi:10.1016/j.breast.2022.08.002)
Supplement: Multimedia component 1 [file mmc1.docx]

**Supplementary Table 1. EORTC QLQ-C30 scores in Full Analysis Set**

|  | N | Mean | SD | Difference (C3D1-C1D1)  Mean (SD) | Difference (EOT-C1D1)  Mean (SD) | T | P-value* |
| --- | --- | --- | --- | --- | --- | --- | --- |
| **Global health status** | | | | | | | |
| C1D1 | 109 | 53.98 | 22.53 |  |  | - | - |
| C3D1 | 87 | 58.72 | 21.45 | 2.30 (23.35) | - | 0.92 | 0.3611 |
| EOT | 85 | 48.24 | 22.57 | - | -6.57 (24.64) | -2.46 | 0.0160 |
| **Functional scales** | | | | | | | |
| C1D1 | 109 | 73.70 | 16.70 |  |  |  |  |
| C3D1 | 87 | 73.21 | 16.68 | -1.17 (13.87) |  | 1.13 | 0.1361 |
| EOT | 85 | 66.12 | 21.38 |  | -8.58 (17.32) | -4.56 | <.0001 |
| **Symptom scales** | | | | | | | |
| C1D1 | 109 | 25.93 | 17.71 |  |  |  |  |
| C3D1 | 87 | 26.85 | 17.32 | 2.51 (11.43) |  | 1.44 | 0.0569 |
| EOT | 85 | 31.95 | 18.82 |  | 6.43 (12.66) | 4.61 | <.0001 |

EOT, end of treatment; SD, standard deviation; * Paired t-test

**Supplementary Table 2. Patient characteristics of the control group in TH3RESA trial and those of this study**

|  | TH3RESA trial (control group)  N=198 | KM10B  N=109 |
| --- | --- | --- |
| Age | 54 (28-85) | 55 (20-76) |
| HR positive/negative | 52%/43% | 54%/44% |
| ECOG PS |  |  |
| 0 | 41% | 0 |
| 1 | 51% | 29.4% |
| 2 | 8% | 70.6% |
| Visceral meta | 76% | Lung 68.8%/ Liver 37.6% |
| Brain meta | 14% | 19.3% |
| Previous anti-HER2 therapy |  |  |
| Trastuzumab | 100% | 99.1% |
| Lapatinib | 100% | 74.3% |
| T-DM1 | 0 | 98.2% |
| Pertuzumab | 0 | 41.3% |
| T-DXd | 0 | 11.0% |
| Number of prior chemotherapies at metastatic setting |  |  |
| ≤ 3 | 39% | 39.4% |
| >3 | 61% | 60.4% |

HR, hormone receptor; PS, performance status; T-DXd, trastuzumab deruxtecan.

**Supplementary Table 3. TPC regimens of the control group in TH3RESA trial and those of this study**

|  | TH3RESA trial (control group)  N=126 (Trastuzumab+chemotherapy) | KM10B  N=109 |
| --- | --- | --- |
| Vinorelbine | 42.9% | 29.4% |
| Gemcitabine | 19.0% | 6.4% |
| Eribuline | 9.5% | 48.6% |
| Paclitaxel | 10.3% | 0 |
| Nab-paclitaxel | 1.6% | 15.6% |
| Capecitabine | 2.4% | 0 |
| Docetaxel | 6.3% | 0 |
| Others (carboplatin, doxorubicin, Cyclophosphamide etc..) | 7.9% | 0 |

TPC, treatment of physician’s choice
